# TLE4 promotes colorectal cancer progression through activation of JNK/c-Jun signaling pathway

**DOI:** 10.18632/oncotarget.6694

**Published:** 2015-12-20

**Authors:** Shu-Yang Wang, Ke Gao, Dan-Ling Deng, Juan-Juan Cai, Zhi-Yuan Xiao, Liu-Qing He, Hong-Li Jiao, Ya-Ping Ye, Run-Wei Yang, Ting-Ting Li, Li Liang, Wen-Ting Liao, Yan-Qing Ding

**Affiliations:** ^1^ Department of Pathology, Nanfang Hospital, Southern Medical University, Guangzhou, Guangdong, China; ^2^ Department of Pathology, School of Basic Medical Sciences, Southern Medical University, Guangzhou, Guangdong, China; ^3^ State Key Laboratory of Oncology in Southern China, Department of Experimental, Guangzhou, Guangdong, China; ^4^ Department of Pathology, The Fifth Affiliated Hospital of Southern Medical University, Guangzhou, Guangdong, China

**Keywords:** TLE4, colorectal cancer, JNK, c-Jun, proliferation

## Abstract

The Groucho transcriptional co-repressor TLE4 protein has been shown to be a tumor suppressor in a subset of acute myeloid leukemia. However, little is known about its role in development and progression of solid tumor. In this study, we found that the expression of TLE4 in colorectal cancer (CRC) tissues was significantly higher than that in their matched adjacent intestine epithelial tissues. In addition, high expression of TLE4 was significantly correlated with advanced Dukes stage, lymph node metastasis and poor prognosis of CRC. Moreover, enforced expression of TLE4 in CRC cell lines significantly enhanced proliferation, invasion and tumor growth. On the contrary, knock down of TLE4 repressed cell proliferation, invasion and tumor growth. Furthermore, our study exhibited that the TLE4 promoted cell proliferation and invasion partially via activation of JNK-c-Jun pathway and subsequently increased cyclinD1 and decreased P27Kip1 expression. In conclusion, these results suggested that TLE4, a potential prognostic biomarker for CRC, plays an important role in the development and progression of human CRC.

## INTRODUCTION

Colorectal cancer (CRC) is one of the most common types of malignant tumor with high morbidity and mortality. The initiation and progression of CRC is a complicated network with multiple genetic and epigenetic genetic changes [1]. It has been well documented that several key signaling pathways were activated via mutational inactivation of tumor suppressors and mutational activation of oncogenes. For examples, mutation in APC or β-Catenin results in activation of canonical Wnt pathway. Mutational activation of KRAS leads to activation of PI3K/AKT, ERK, and NF-κB pathways [2, 3]. Despite the early diagnosis and advanced treatments in recent years, the clinical outcome and prognosis of CRC patients remain pessimistic. Efforts to elucidate newer biomarkers and more effective therapies are still imperative in order to improved survival for CRC patients [4].

The Groucho (Gro)/TLE protein belongs to a large family of co-repressors that are globally expressed and highly conserved from yeast to human [5]. Proteins in the Groucho family can regulate transcription by either direct binding to a variety of DNA-binding transcription factors or recruitment of histone deacetylases and methylases to form large multi-protein complexes [6]. Groucho proteins act in key developmental signaling pathways including receptor tyrosine kinase/Ras/MAPK, Notch, Wnt, as well as Hedgehog, and play essential roles in diverse processes during embryonic development and morphogenesis [5, 7, 8]. The major downstream genes affected by the Groucho family include members of the Hes, Runx, LEF1/Tcf, Pax, and Myc families, which are key regulators during hematopoiesis and leukemogenesis [9]. A number of Groucho proteins have been identified in a variety of eukaryotic organisms, including invertebrate and vertebrate species [9]. The human genome encodes at least six Groucho family members, termed transducin-like enhancer of Split 1-6 (TLE1-6) [10]. Besides their essential functions implicated in embryonic development, emerging researches have revealed the roles of Groucho family members in human cancer. For instance, TLE1 was overexpressed in a significant number of human lung cancer tissues and was implicated to be a putative lung specific oncogene in a transgenic mice model [11]. Ectopic expression of TLE1 promoted EMT by suppressing E-cadherin in lung cancer cells [12]. Another Groucho family member TLE3 was found to be associated with the sensitivity to taxane treatment for ovarian carcinoma and breast cancer [13, 14]. TLE4 played an important role in the extrinsic and intrinsic regulation of hematopoiesis and in bone development [15]. In addition, TLE4 acts as a tumor suppressor gene in acute myeloid leukemia. Knockdown of TLE4 in AML1-ETO-expressing Kasumi-1 cell line increased cell division, whereas forced expression of TLE4 caused apoptosis and cell death [16]. However, little is known about its function in solid cancer.

Since it has been well documented that most colorectal cancers harbor APC or β-catenin mutations [17]. In addition, TCF/LEF-mediated gene transcription may depend on a balance between β-catenin and Groucho proteins. Thus, it will be interesting to investigate the expression and role of Groucho factors in human colorectal cancers. In the present study, we aim to investigate the expression pattern and potential role of TLE4 in the development and progression of CRC. We found that TLE4 expression is significantly higher in CRC tissues than matched non-tumor mucosa tissues. In addition, the high expression level of TLE4 is significantly correlated with aggressive characteristics and poorer overall survival of CRC patients. Moreover, TLE4 could promote cell proliferation and tumor growth invasion in CRC partially through acceleration of JNK/c-Jun pathway.

## RESULTS

### The expression of TLE4 was up-regulated in CRC

**Figure 1:**
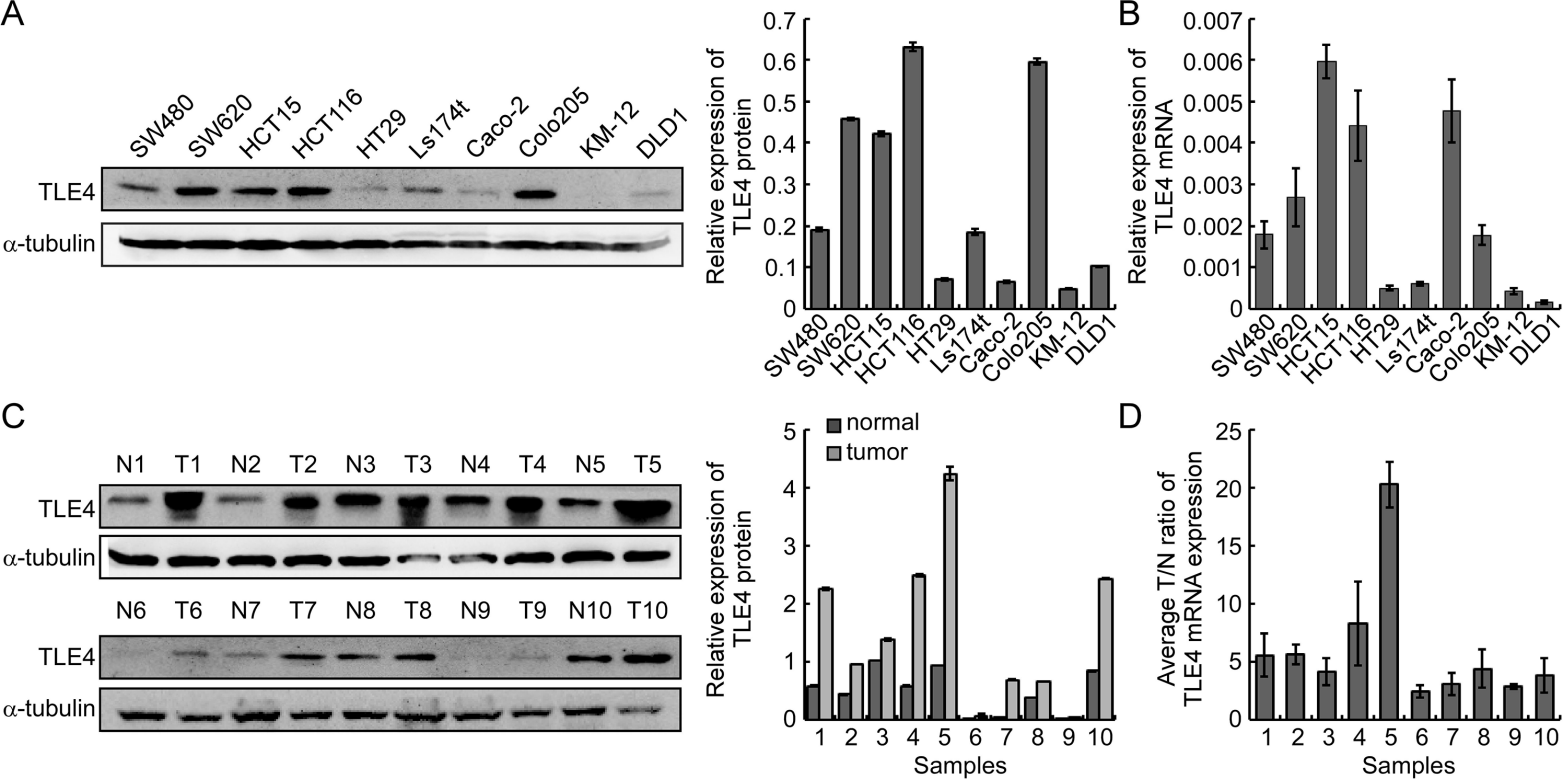
TLE4 expression is evaluated in CRC cell lines and primary human CRC (**A**) Detection of TLE4 protein expression by western blotting in ten CRC cell lines (left). The gray level of each band was compared by Quantity One Software to test the protein expression levels (right). (**B**) Real-time PCR of TLE4 mRNA expression in ten CRC cell lines. Error bars represent mean ± SD calculated from 3 parallel experiments. (**C**) Western blotting of TLE4 expression in 10 paired human CRC tissues (T) and the matched adjacent non-tumor tissues (N) from the same patient (left). The gray level of each band was compared by Quantity One Software to test the protein expression levels (right). (**D**) Real-time PCR was used to quantify average T/N ratios of TLE4 expression. Error bars represent mean ± SD calculated from 3 parallel experiments. The expression levels of protein or mRNA were normalized with α-tubulin or GAPDH.

Real-time PCR and Western Blotting analysis were utilized to test the expression of TLE4 in 10 CRC cell lines, including SW480, SW620, HCT15, HCT116, Ls174t, HT29, Caco-2, Colo205, KM12 and DLD1. Our results revealed that TLE4 was differently expressed in all the 10 CRC cell lines. (Figure 1A, 1B). In addition, the expression of TLE4 protein and mRNA was significantly up-regulated in ten CRC tissue (T) compared with their paired adjacent normal intestine epithelial tissue (N) (Figure 1C, 1D).

### Up-regulation of TLE4 is associated with progression and poor prognosis in CRC

**Figure 2:**
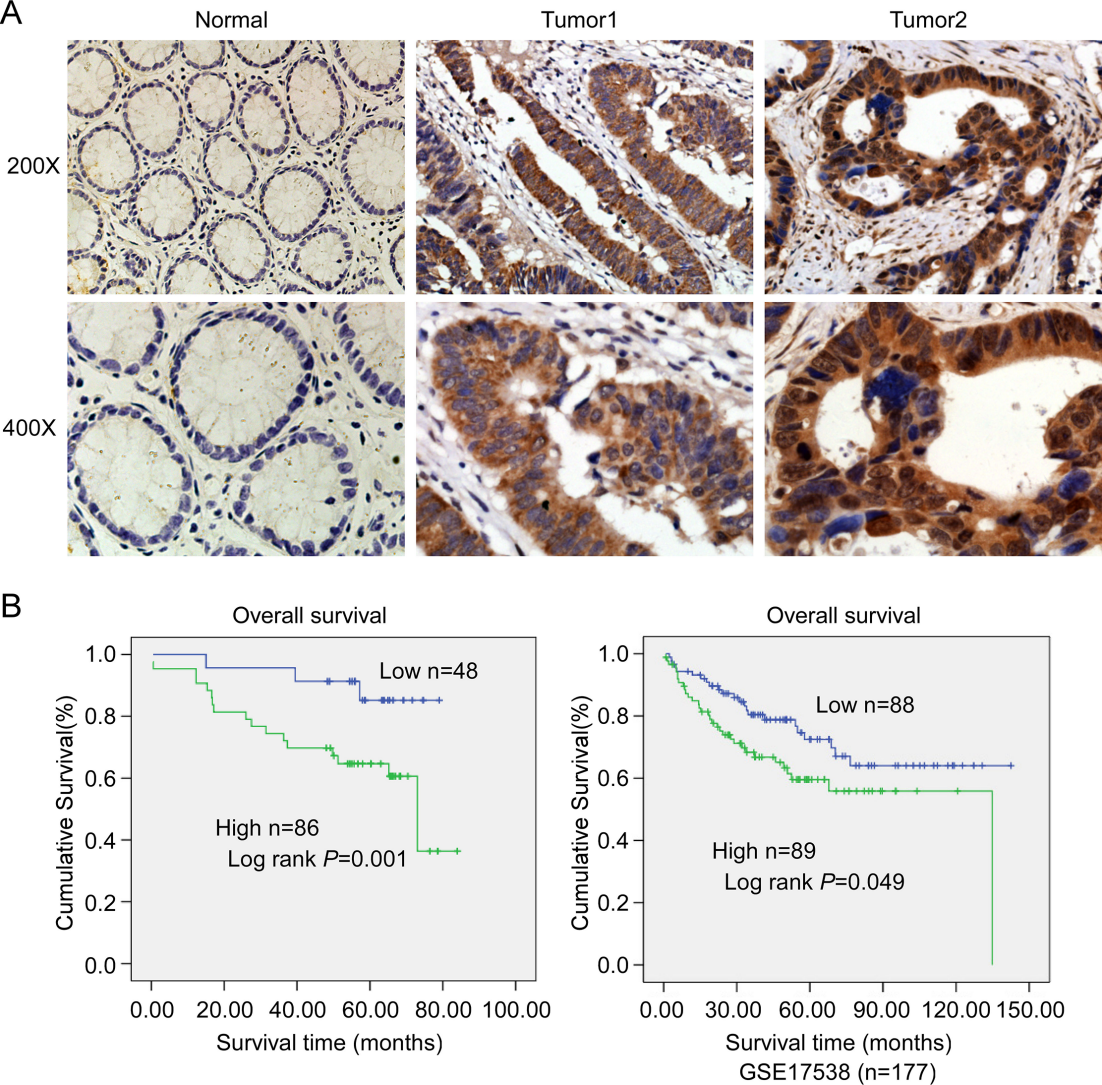
Expression of TLE4 was associated with progression and poor prognosis in CRC (**A**) Representative images of TLE4 expression in normal intestinal epithelium and CRC specimens examined by IHC. TLE4 was positively detected in CRC cells (middle and right), whereas it was only weakly (left) detected in normal intestinal epithelium cells. (**B**) Influence of TLE4 expression on overall survival by Kaplan–Meier analysis in 134 CRC patients (left) and 177 CRC patients from a public clinical microarray dataset of GSE17538 [18] (right).

IHC was used to detect the expression level of TLE4 in 134 cases paraffin-embedded CRC tissue sections. The results showed that TLE4 protein located both in the cytoplasm (Figure 2A, middle) and nucleus (Figure 2A, right), and the expression of TLE4 increased markedly in 64% (86/134) CRC tumor tissue (Figure 2A middle and right) compared with that in adjacent non-tumor tissue (Figure 2A left). The correlation between TLE4 expression and clinicopathologic features of CRC was analyzed by Mann-Whitney *U* tests. As summarized in Supplementary Table 1, the results exhibited that the high expression level of TLE4 significantly was associated with poor Dukes stage (*P* = 0.001) and more lymph node metastasis (*P* = 0.001). Spearman correlation analysis was further used to confirm these data (Supplementary Table 2), and the coefficients for the correlations between TLE4 expression and Dukes stage and lymph node metastasis were 0.506 (*P* < 0.001) and 0.421 (*P* < 0.001), respectively. The result of Kaplan-Meier survival analysis also indicated that patients with high TLE4 expression levels had a poor prognosis in 134 CRC patients (Figure 2B left) and 177 CRC patients from a public clinical microarray database of GSE17538 [18] (Figure 2B right). Cox regression analyses revealed that lymph node metastasis and TLE4 expression were recognized as independent prognostic factors in this study (Supplementary Table 3).

### Overexpression of TLE4 promoted proliferation, invasion and tumorigenesis of CRC cells

**Figure 3:**
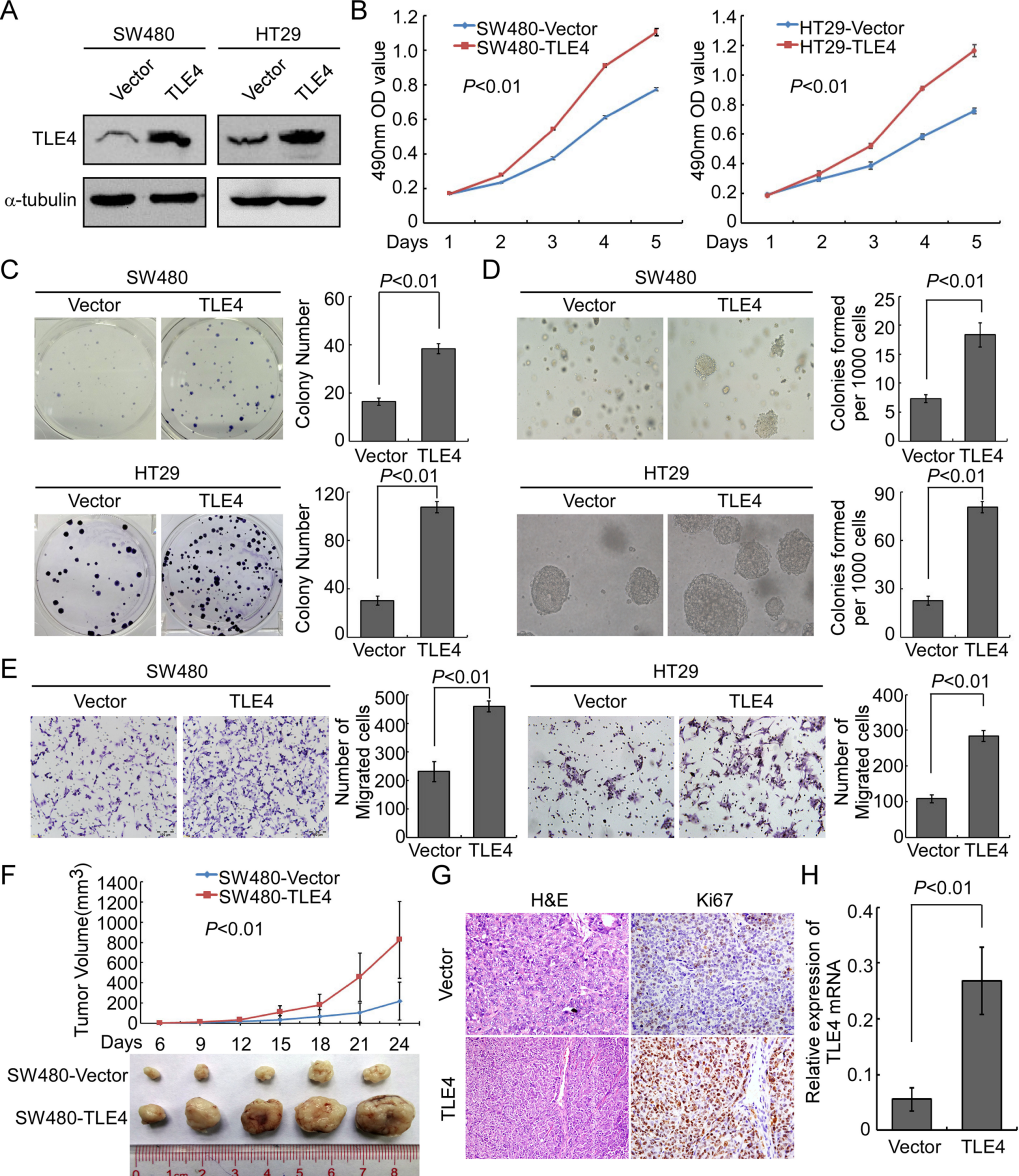
Up-regulation of TLE4 promotes cell proliferation and invasion activity of CRC cells (**A**) Overexpression of TLE4 in SW480 and HT29 cells analyzed by Western blotting. α-Tubulin was used as a loading control. (**B** and **C**) Overexpression of TLE4 promotes SW480 and HT29 cell proliferation in MTT assays (B) and colony formation assays (C). (**D**) Ectopic expression of TLE4 promotes anchorage independent growth ability of SW480 and HT29 cells as determined by Soft agar assays. Colonies containing more than 50 cells were scored. Each bar represents the mean ± SD of 3 independent experiments. (**E**) The invasive abilities of CRC cells evaluated using the Matrigel-coated Boyden chamber invasion assay. Each bar represents the mean ± SD of three independent experiments (**F**) Xenograft model was built by injected SW480/Vector and SW480/TLE4 cells in nude mice (*n* = 5/group). Tumor volumes were measured on the indicated days. Data points are the mean tumor volumes ± SD. (**G**) The sections of tumor were under H & E staining or subjected to IHC staining using an antibody against Ki-67. (**H**) The expression of TLE4 in xenograft tumor was analyzed with Real-time PCR. Error bar represents the mean ± SD.

In order to explore the possible role of TLE4 in the development and progression of CRC, stable TLE4 expressed cell lines SW480-TLE4, HT29-TLE4 and SW620-TLE4 were made (Figure 3A and Supplementary Figure 1A). The results of MTT assay and colony formation assay showed that TLE4 overexpression promoted the proliferation of SW480, HT29 and SW620 cells compared with control cells (Figure 3B, *P* < 0.01; Figure 3C, *P* < 0.01; Supplementary Figure 1B and 1C, *P* < 0.01). We also examined the effect of TLE4 overexpression on the anchorage-independent growth activity of CRC cells using soft agar formation assays. The results showed that TLE4 overexpression accelerated the proliferation of SW480, HT29 and SW620 cells in soft agar and formed more colonies in comparison with control cells (Figure 3D, Supplementary Figure 1D; *P* < 0.01). Furthermore, overexpression of TLE4 significantly enhanced the invasive ability of CRC cells *in vitro*, as evaluated by Matrigel-coated Boyden chamber invasion assay (Figure 3E, Supplementary Figure 1E; *P* < 0.01). We next detected the effect of TLE4 overexpression on tumor growth using the nude mice xenograft model *in vivo*, using SW480-TLE4 and control cells. As shown in Figure 3F, in comparison with control cells, SW480-TLE4 cells implanted in nude mice exhibited more rapid growth speed and significantly larger tumor volumes (*n* = 5; *P* < 0.01). In addition to the difference of tumor volume, we also found that the tumors formed by SW480-TLE4 cells displayed a higher Ki-67 index than that in tumors formed by SW480-Vector cells, as detected by IHC analysis of Ki-67 (Figure 3G).

### Knocking down of TLE4 inhibited proliferation, invasion and tumorigenesis of CRC cells

**Figure 4:**
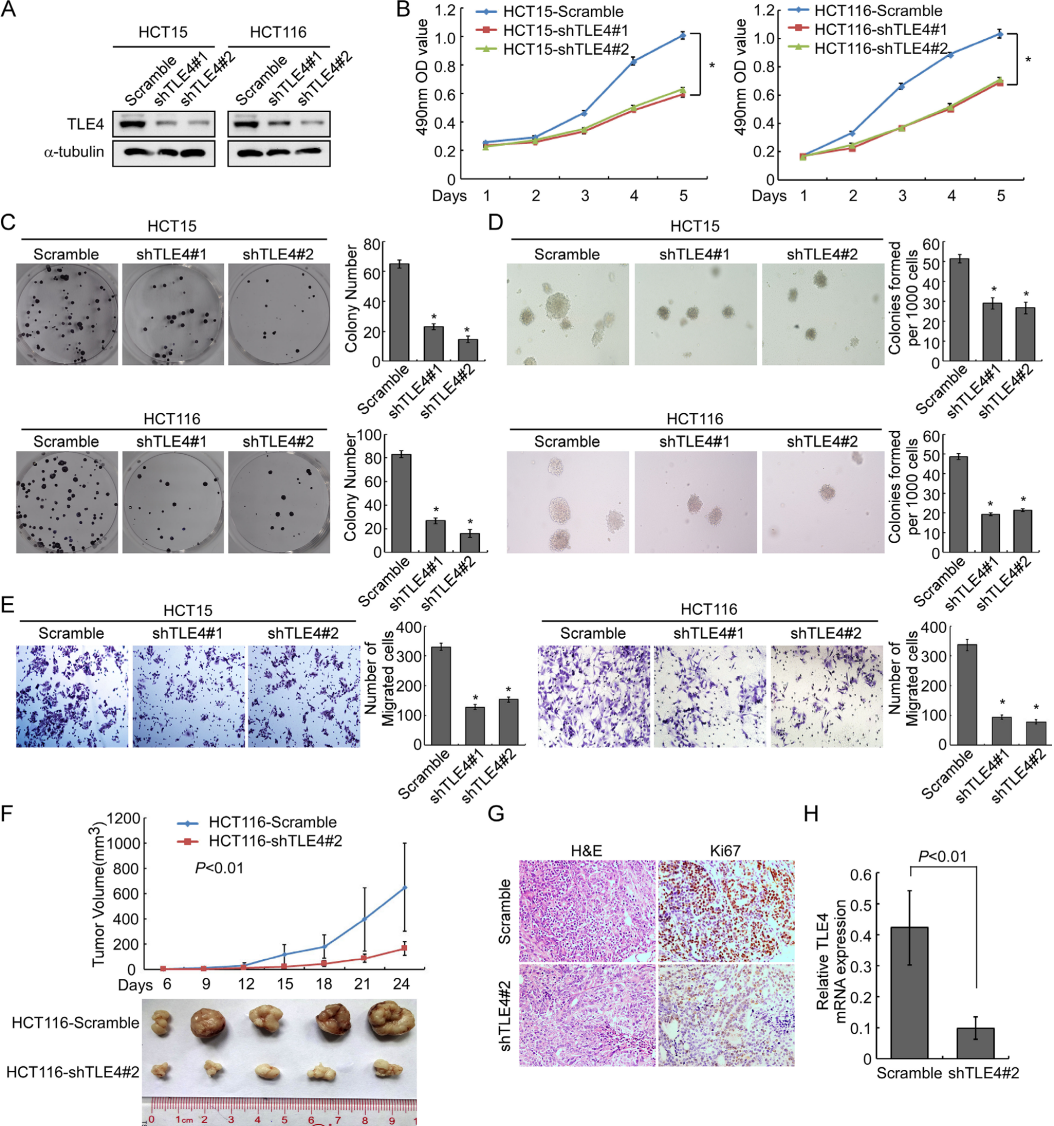
Depletion of TLE4 inhibits cell proliferation and invasion activity (**A**) RNAi-silencing of TLE4 in shRNA-transduced stable HCT15 and HCT116 cells. α-Tubulin was used as a loading control. (**B** and **C**) Reduction of endogenous TLE4 inhibited cell growth in MTT assays (B) and colony formation assays (C) *P < 0.01. (**D**) Silencing of TLE4 inhibited cell growth ability of HCT15 and HCT116 in Soft agar colony formation assays. Colonies containing more than 50 cells were scored. Error bar represents the mean ± SD of three independent experiments *P < 0.01. (**E**) The invasive abilities of CRC cells evaluated using the Matrigel-coated Boyden chamber invasion assay. Each bar represents the mean ± SD of three independent experiments. *P < 0.01. (**F**) Xenograft model was built by injected HCT116/vector and HCT116/shTLE4 cells in nude mice (*n* = 5/group). Tumor volumes were measured on the indicated days. Data points are the mean tumor volumes ± SD. (**G**) The sections of tumor were under H & E staining or subjected to IHC staining using an antibody against Ki-67. (**H**) Real-time PCR was used to test TLE4 expression in xenograft tumors formed from HCT116/Scramble and HCT116/shTLE4. Error bar represents the mean ± SD.

To further confirm the impact of TLE4 on proliferation, invasion and tumorigenesis of CRC cells, we knockdown endogenous TLE4 in HCT15 and HCT116 CRC cells using shRNAs specifically targeting TLE4 (Figure 4A). The results of MTT assay and colony formation assay demonstrated that silence of TLE4 expression caused obviously reduced cell growth in HCT15 and HCT116 cells as compared with control cells (Figure 4B and 4C; *P* < 0.01). We next detected the effect of TLE4 silencing on the anchorage-independent growth activity with soft agar assay. The result demonstrated that depletion of endogenous TLE4 in HCT15 and HCT116 cells caused significant decreasing in colony number and colony size in soft agar (Figure 4D; *P* < 0.01). Migration assay showed that silence of TLE4 inhibited invasive ability of HCT15 and HCT116 cells (Figure 4E; *P* < 0.01). *In vivo* tumorigenesis assay exhibited that knockdown of endogenous TLE4 expression in HCT116 cells caused significant inhibition of tumor growth (Figure 4F; *n* = 5; *P* < 0.01). IHC staining showed that the tumors of control group displayed much higher Ki-67 index than that in HCT116-shTLE4 (Figure 4G).

### Activation of JNK/c-Jun signaling pathway was involved in TLE4-mediated acceleration of proliferation and invasion of CRC cells

**Figure 5:**
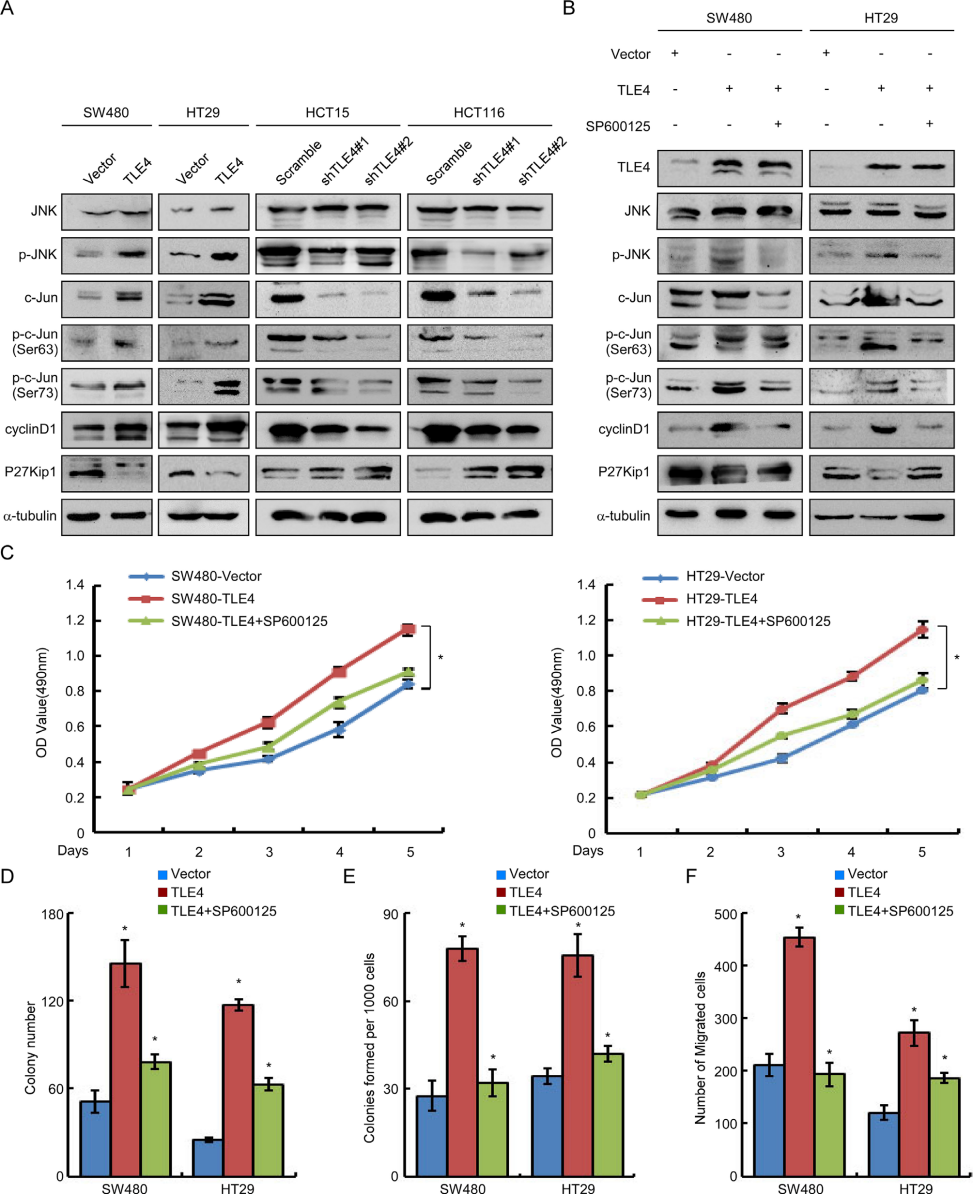
TLE4 activates JNK-c-Jun pathway in CRC cells (**A**) TLE4 increases the JNK-c-Jun pathway activity and expression of cyclin D1 and P27Kip1 in CRC cells. (**B**) SW480/TLE4 and HT29/TLE4 cells were treated with JNK inhibitor SP600125 (10 μM) for 24 h. Inhibition of the JNK signaling inhibits the promoting effect of TLE4-overexpression on JNK-c-Jun activity in CRC cells. (**C**, **D**, **E** and **F**) Inhibition of the JNK signaling blocks the promoting effect of TLE4-overexpression on cell proliferation and invasion of CRC cells as determined by MTT assay (C), colony formation assay (D), soft agar assay (E) and migration assay (F) after treatment with SP600125 (10 μM). Error bars represent mean ± SD from 3 independent experiments; *P < 0.01.

Finally, we explored the possible mechanism of TLE4 accelerating proliferation and invasion of CRC cells. We observed that overexpression of TLE4 dramatically enhanced the expression levels of c-Jun, p-c-Jun (Ser-63/73) and p-JNK in SW480, HT29 and SW620 cells (Figure 5A and Supplementary Figure 2A, left). On the contrary, knock down of TLE4 expression in HCT15 and HCT116 cells significantly decreased c-Jun, p-c-Jun and p-JNK levels (Figure 5A). Furthermore, we examined the expression of representatives of c-Jun target genes cyclinD1 and P27Kip1. The result demonstrated the increased expression of cyclinD1 and decreased expression of P27Kip1 in TLE4-overexpressing cells. In contrast, significant increases in the expression of P27Kip1 and decreases of cyclin D1 were shown in TLE4 knockdown CRC cells.

To further validate whether TLE4 accelerated proliferation and invasion of CRC cells by activating JNK signaling pathway, we treated TLE4 overexpression CRC cells with a specific JNK inhibitor (SP600125) [19]. As shown in Figure 5B and Supplementary Figure 2A (right), blocking JNK activity by SP600125 largely diminished the increase of p-JNK and p-c-JUN levels in SW480, HT29 and SW620 cells with TLE4 overexpression. Moreover, we detected the effects of TLE4 on cell proliferation and invasion in TLE4 overexpression cells treated with or without SP600125 to block JNK pathway. The results of MTT, colony formation, soft agar and migration assays showed that the proliferation and invasion of TLE4 overexpression cells was significantly compromised by treatment with the JNK inhibitors (Figure 5C–5F; Supplementary Figures 2B–2E and 3).

## DISCUSSION

TLE4 belongs to a highly conserved transcriptional co-repressor family and was believed to be a tumor suppressor gene in acute myeloid leukemia [16]. However, little is known about its role in solid cancer. In this study, we presented the first evidence of TLE4 up-regulation in CRC biopsies at both mRNA and protein levels as compared with adjacent non-cancerous tissues. High expression of TLE4 protein was significantly correlated with aggressive characteristic and poor prognosis of patients. These results suggest that up-regulation of TLE4 protein expression might act as a biomarker to identify patients with poorer outcome and aggressive CRC.

There were two opposite functions of TLEs in different contexts. TLE1 and TLE4 were considered as tumor suppressors in hematological malignancy. Both of TLE1 and TLE4 localize in chromosome 9q, the commonly deleted region in acute myeloid leukemia (AML) [20]. Low levels of TLE1 and TLE4 expression in myeloid cell lines and subsets of AML samples were observed. Knockdown of TLE1 or TLE4 levels increased the rate of cellular proliferation in AML1-ETO expressing cells, while forced expression of either TLE1 or TLE4 caused apoptosis and cell death [16]. However, a series of independent findings indicate a survival promoting role of TLE1 in solid cancer. TLE1 overexpression stimulated anchorage-independent growth in chicken embryo fibroblast [21] and promoted epithelial-mesenchymal transition in lung cancer cells [12]. Recently, it was reported that TLE1 is a putative lung specific oncogene as revealed in a transgenic mouse model [11]. In addition, TLE1 was overexpressed in a significant number of human lung cancer tissues, including squamous cell carcinomas and adenocarcinomas [11]. TLE1 and TLE4 are both full-length Groucho proteins possessing all the domains of the prototype, including two remarkably conserved N- and C-terminal domains and three less conserved internal regions [10, 22], suggesting that these two proteins may share similar function in some aspects. In this study, TLE4 was shown to be able to promote proliferation, invasion and tumor growth in CRC, both *in vitro* and *in vivo*. This result provides evidence that TLE4 functions distinctively in solid tumor from hematological malignancy.

Although aberrant expression of TLE4 has been implicated in tumorigenesis, the underlie mechanism remains largely unknown. It has been documented that Groucho family proteins repress Wnt pathway by binding with TCF/LEF1 complex. However, in our study, we didn't observe the changes of Wnt signaling pathway activity (data not shown). Moreover, consistent with other research, we observed that TLE4 localized both in the nuclear and cytoplasm in CRC tissues. These results suggested that the role of Groucho protein on suppression of TCF/LEF1 complex might be context dependent. Alternative molecular mechanism might exist in TLE4 mediated acceleration of progression in CRC. Recently, it was reported that TLE4 could repress P27Kip1 with Cux1 during kidney development [23]. P27Kip1 is associated with the progression and outcomes of diverse malignancies including CRC [24–30] and can be directly regulated by JNK/c-Jun at transcriptional level and posttranslational levels [31–33]. The oncogene c-Jun is frequently activated in various cancers to promote cell proliferation and tumor growth [34, 35]. Activation of JNK is also essential for the modulation of cell proliferation and cell motility in human cancers [36–38]. Therefore, we tried to explore whether TLE4 could active JNK/c-Jun pathway and repress P27Kip1 in CRC. The results from this study demonstrated that TLE4 can activate c-Jun and JNK in colorectal cancer cells, and followed by alteration of their downstream targets cyclinD1 and P27Kip1 [31–33]. In addition, blocking JNK activity could largely reverse the increased proliferation and invasion mediated by TLE4, suggesting that JNK/c-Jun signaling was involved in the TLE4 mediated acceleration of CRC progression. However, the underlying mechanism through which TLE4 could activate the JNK/c-Jun signaling needs further investigation.

In summary, our findings suggested that up-regulation of TLE4 might be a valuable prognostic marker of CRC progression. Up-regulation of TLE4 might be important for development and progression of CRC, partially through regulation of JNK/c-Jun pathway. Our study also helps provide evidence for diverse molecular mechanism by which TLE4 is able to promote tumorigenesis of CRC. However, the underlying mechanism need further investigation in detail.

## MATERIALS AND METHODS

### Cell cultures

The human CRC cell lines (SW480, SW620, HCT15, HCT116, Ls174t, HT29, Caco-2, Colo205, KM-12 and DLD1) were initially purchased from American Type Culture Collection (Manassas, VA, USA). SW620 and HT29 were cultured in DMEM medium (Invitrogen, Carlsbad, CA, USA) with 10% FBS (Gibco). SW480, HCT15, HCT116, Ls174t, Caco-2, Colo205, KM-12 and DLD1 were cultured in RPMI 1640 medium (Invitrogen, Carlsbad, CA, USA) containing 10% FBS (Gibco).

### Patients and specimens

This study was conducted on a total of 134 archived, formalin-fixed paraffin-embedded human colorectal carcinoma specimens, which were obtained from the Department of Pathology, NanFang hospital, Southern Medical University, China. All of these cases were clinically and histologically diagnosed between 2000 and 2005. The stage of disease was determined according to the tumor size, lymph node involvement and distant metastasis (pTNM) classification system [39]. The patients are consisted of 71 males and 63 females, ranging in age from 27 to 76 years-old (mean, 56 years). The median follow-up time for overall survival was 73 months for patients still alive at the time of analysis (ranged 0.5 to 79 months). A total of 42/134 (31%) patients died during follow-up. The 10 cases of fresh colorectal cancer tissue were collected in 2014 at the Department of Pathology, Southern Medical University, and CRC tissues as well as the paired adjacent normal tissues were freshly frozen in liquid nitrogen and stored at −80°C until further use. The fresh CRC tissue and paired normal mucosal tissue specimens taken from sites distant to the cancerous lesion were obtained from CRC patients who had undergone surgical resection, and then stored in liquid nitrogen until further use.

### Vectors construction and retroviral infection

The TLE4 construct was generated by subcloning PCR amplified full-length human TLE4 cDNA into pBabe. For silencing of TLE4, 2 short hairpin RNA (shRNA) sequence was cloned into the pGPU6/GFP/Neo vector to generate pGPU6/GFP/Neo-RNAi(s), respectively. Retroviral production and infection were performed as previously describe [40]. Stable cell lines expressing TLE4 or shTLE4 were respectively selected for 10 days with 0.5 mg/mL puromycin or 1 mg/mL G418.

### Immunohistochemistry

Immunohistochemistry (IHC) staining and scoring were done as previously described [41]. For details, please see the Supplementary Methods.

### Real-time RT-PCR and Western blotting analyses

Total RNA extraction and real-time RT-PCR were performed as previously described, using the ABI PRISM 7500 Sequence Detection System (Applied Biosystems) [42]. For details, please see the Supplementary Methods.

### MTT assay, colony formation assay, soft agar assay and migration assay

MTT assay, Colony formation assay, Soft agar assay and Migration assay were performed as previously described [41, 43]. For details, please see the Supplementary Methods.

### Tumorigenesis in nude mice

Xenograft models were formed by subcutaneous injection of CRC cells (2 × 106), including SW480-Vector and SW480-TLE4, HCT116-Scramble and HCT116-TLE4 RNAi#2 (*n* = 5 for each group), on the hind limbs of 4–6 week-old Balb/C athymic nude mice (nu/nu) achieved from Animal Center of Southern Medical University, Guangzhou, China. All mice were raised and fed under SPF conditions, and all experiments were under the approvement of the Use Committee for Animal Care and proceeded on the basis of institutional guidelines. Tumor size was measured with a slide caliper and tumor volume was calculated by the formula 0.44 × A × B^2^ (A represents the base diameter of tumor and B represents the corresponding perpendicular value). The tumors were excised, then fixed with 10% neutral buffered formalin and 4 μm sections were cut. The sections were stained with hematoxylin and eosin according to standard protocols, then further under IHC staining using antibody against Ki-67.

### Statistical analysis

SPSS version 13.0 was used for all statistical analyses. Mann-Whitney *U* tests were utilized to analyze the correlation between the clinicopathologic features of CRC and TLE4 expression. Survival curves were plotted by the Kaplan-Meier method and compared using the log-rank test. *P* < 0.05 was considered significant.

### Accession numbers for data sets

The clinical data sets generated and reanalyzed in the study came from the GEO database (GSE17538).

## SUPPLEMENTARY FIGURES AND TABLES


