# Supplementary material for: TLE4 promotes colorectal cancer progression through activation of JNK/c-Jun signaling pathway
Source: Oncotarget. 2015 Dec 20;7(3):2878–88. doi: 10.18632/oncotarget.6694 (PMC4823078; doi:10.18632/oncotarget.6694)
Supplement: Supplementary file 1 [file oncotarget-07-2878-s001.pdf]

TLE4 promotes colorectal cancer progression through activation of JNK/c-Jun signaling pathway

Supplementary Materials

SUPPLEMENTARY METHODS

Immunohistochemistry

The sections were reviewed and scored respectively by two observers. Observational index includes two aspects, the proportion of positively stained tumor cells and the strength of staining. The ratio of positive tumor cells was according to the following standards graded as follows: 0 (no positive tumor cells), 1 (less than 10% positive tumor cells), 2 (10–50% positive tumor cells), and 3 (more than 50% positive tumor cells). The strength of staining was scored as following criteria: 0 (no staining); 1 (weak staining = light yellow), 2 (moderate staining = yellow brown), and 3 (strong staining = brown). The staining index (SI) was counted as staining strength score x proportion of positive tumor cells. Using this method of estimation, the expression of TLE4 was scored as 0, 1, 2, 3, 4, 6 and 9. Critical values for TLE4 were chosen in accordance with a measure of heterogeneity by the log-rank test statistical analysis in regard to overall survival. An optimal critical value was identified: ≤ 3 as low expression of TLE4, and the score of ≥ 4 was used to define tumors as high TLE4 expression.

Real-time RT-PCR and Western blotting analyses

Total RNA extraction and real-time RT-PCR were performed using the ABI PRISM 7500 Sequence Detection System (Applied Biosystems). Real-time PCR primers were designed using the Primer Express. Sequences of the real-time PCR primers were: TLE4 sense, 5′-GTTTCCGAGGTGCTGAGAAG-3′; anti-sense, 5′-TAATCGGGGCATCTTTCTTG-3′; GAPDH, sense, 5′-GACTCATGACCACAGTCCATGC-3′; anti-sense, 5′-AGAGGCAGGGATGATGTTCTG-3′. Reverse Transcription was carried out with the PrimeScript™ RT-PCR Kit for RT-PCR (Invitrogen, Carlsbad, CA) according to the manufacturer's protocol. Real-time RT-PCR was carried out using SYBR Green I (Applied BioSystems, Foster, CA). The data were normalized to the geometric mean of housekeeping gene GAPDH and calculated as 2-DDCT method.

Western blotting was performed according to standard methods as described previously [44], using anti-TLE4 (Abcam Plc, USA), anti-p-JNK, anti-JNK, anti-P27Kip1 (Bioworld Technology Inc. St. Louis Park, MN, USA), anti-cyclin D1 (BD Pharmingen), anti-c-Jun at Ser-63,anti-c-Jun at Ser-73 and anti-c-Jun (Cell Signaling Technology, Danvers, MA, USA). A mouse monoclonal anti-α-Tubulin antibody (Sigma, Saint Louis, MO, USA) was used as loading control.

MTT assay

Cell were trypsinized and seeded on 96-well plates (1×10^3), then incubated for 24 hours at 37°C. 20 ul of 5 g/L 3-(4,5-dimethylthiazol-z-yl)-2,5-diphenyltetrazolium bromide (MTT, Sigma, St Louis, MO, USA) was mixed into each well and incubated for 4 hours at 37°C. 150 ul dimethyl sulphoxide (DMSO, sigma, St, Louis, MO, USA) were added into the wells after removing the MTT-medium mixture. The absorption photometric was measured at 490 nm with a Microplate Autoreader (Bio-Rad, Hercules, CA, USA). Each experiment was repeated at least three times.

Colony formation assay

200 cells were planted on 60mm plates and cultured at 37°C in a humid atmosphere of 5% CO2 for 2 weeks. The plates were fixed with 4% paraformaldehyde for 30 minutes after washing with PBS for 3 times. The colonies were stained with 1% crystal violet for 30 s. Colony contained more than 50 cells was counted. Three independent experiments were performed. The data was calculated using Student's *t*-tests.

Soft agar assay

60 mm plates were covered with a layer of 0.66% agar in medium supplemented with 20% fetal bovine serum. Cells (1×10^4) were suspended in 2 ml RPMI 1640 supplemented with 10% fetal bovine serum with 0.33% agar, and were then seeded on the surface of the prepared plates. The plates were cultured at 37°C for 2 weeks and the colonies were gauged with an ocular micrometer. Only colonies that larger than 0.1 mm in diameter were counted. The data was calculated using Student's *t*-tests. Each experiment was repeated at least three times.

Migration assay

Migration assay was performed as previously described [45]. Briefly, cells (5×10^4) in culture medium containing 1% fetal bovine serum were seeded in the upper chamber of Boyden chambers. Culture medium with 10% fetal bovine serum was added in the lower chamber as a chemoattractant. After incubation for 24 h, cells on the upper side of the filter were removed with cotton swabs. Cells that migrated to the lower surface of the filter were fixed in 4% paraformaldehyde and stained with Giemsa. The migratory cells were counted (10 random 200 × fields per well). Three independent experiments were performed and the data were presented as the mean ± SD.

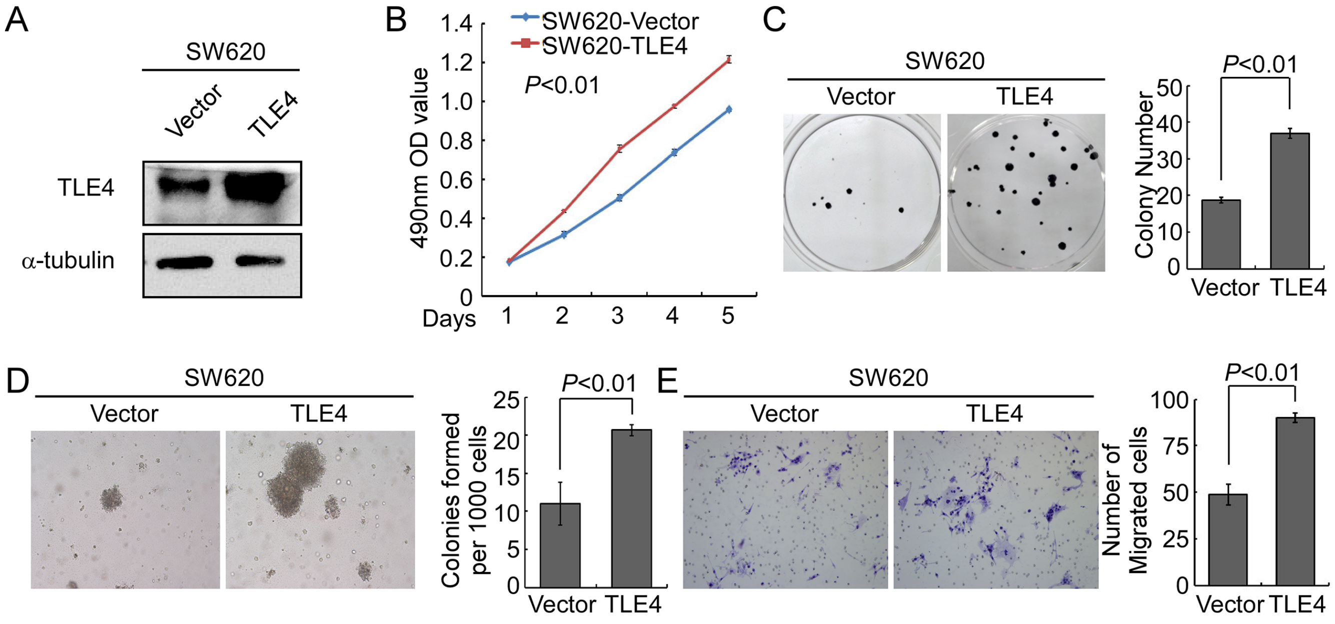

Supplementary Figure S1: TLE4 promotes cell proliferation and invasion in SW620 cells. (**A**) Overexpression of TLE4 in SW620 cells analyzed by Western blotting. α-Tubulin was used as a loading control. (**B**, **C**, **D** and **E**) Overexpression of TLE4 promotes SW620 cells proliferation and invasion in MTT assay (B), colony formation assay (C), soft agar assay (D) and migration assay (E). Each bar represents the mean ± SD of 3 independent experiments.

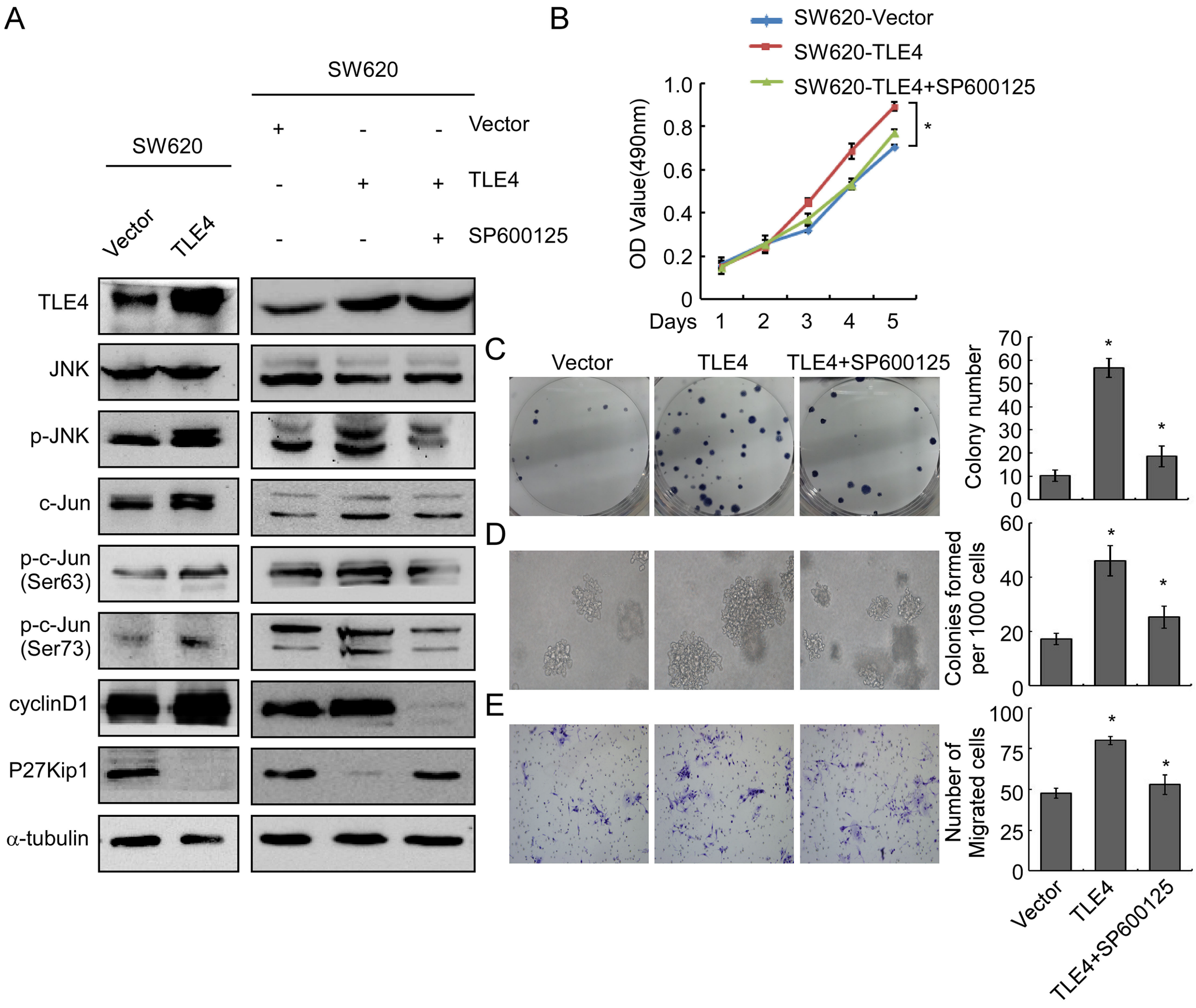

Supplementary Figure S2: TLE4 activates JNK-c-Jun pathway in SW620 cells. (**A**) TLE4 regulates the JNK-c-Jun pathway activity and expression of cyclin D1 and P27Kip1 in SW620 cells (left). Inhibition of the JNK signaling inhibits the promoting effect of TLE4-overexpression on JNK-c-Jun activity in SW620 cells (right). (**B**, **C**, **D** and **E**) Inhibition of the JNK signaling blocks the promoting effect of TLE4-overexpression on cell proliferation and invasion of CRC cells as determined by MTT assay (B), colony formation assay (C), soft agar assay (D) and migration assay (E) after treatment with SP600125 (10 μM). Error bars represent mean ± SD from 3 independent experiments; *$P < 0.01$.

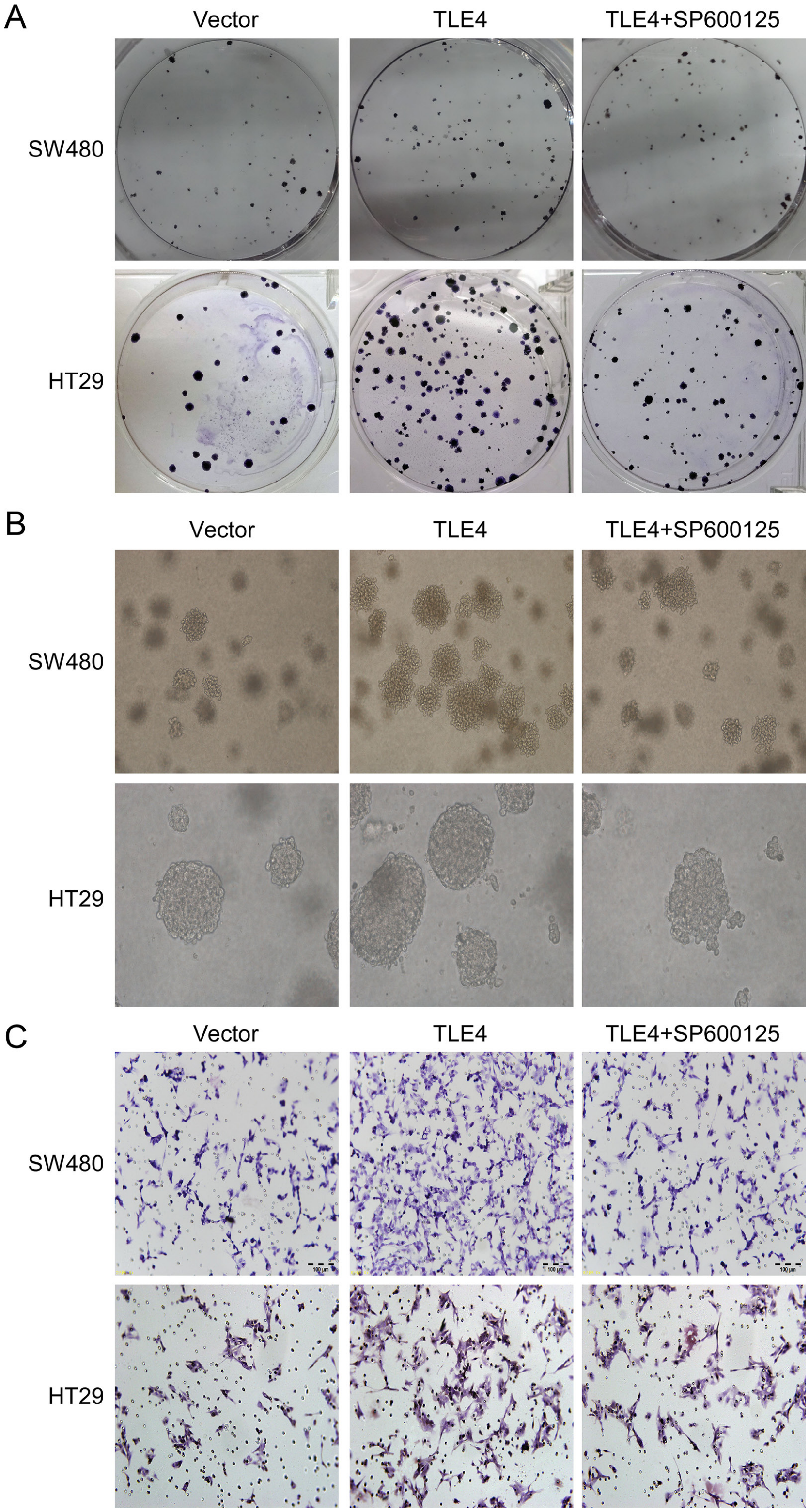

Supplementary Figure S3: Inhibition of the JNK signaling blocks the promoting effect of TLE4-overexpression on cell proliferation and invasion of SW480 and HT29 cells as determined by colony formation assay (A), soft agar assay (B) and migration assay (C) after treatment with SP600125 (10 μM).

Supplementary Table S1: Correlation between clinicopathologic features and TLE4 expression levels

Characteristics	TLE4 levels		*P* values
	Low	High	
**Age**			
≤ mean (56)	24	42	0.520
> mean (56)	24	44	
**Gender**			
Male	26	45	0.491
Female	22	41	
**Differentiation**			
Well	14	12	0.071
Moderate	20	50	
Poor	14	24	
**Dukes stage**			
Dukes A	6	8	0.001
Dukes B	36	22	
Dukes C	4	54	
Dukes D	2	2	
**T stage**			
1	24	38	0.169
2	20	46	
3	2	0	
4	2	2	
**Lymph node involvement**			
No	42	30	0.001
Yes	6	56	
**Distant metastasis**			
No	46	84	0.452
Yes	2	2	

Supplementary Table S2: Spearman correlation analysis between TLE4 and clinicopathologic features

Variables	TLE4 levels	
	Spearman correlation	*P* values
Dukes stage	0.506	< 0.001
Lymph node involvement	0.421	< 0.001

Supplementary Table S3: Multivariate Cox regression analysis of TLE4 levels and other potential prognostic factors for CRC patients

Variable	Category	No.	Multivariate analysis		
			RR	95% CI	*P* value
TLE4 levels	Low	48	2.228	1.040–4.771	0.039
	High	86			
Lymph node involvement	No	72	0.031	0.002–0.410	0.008
	Yes	62			

RR: relative risk; CI: confidence interval.
